# Dysregulated microRNA Expression Relevant to *TERT* Promoter Mutations in Tonsil Cancer—A Pilot Study

**DOI:** 10.3390/life13102090

**Published:** 2023-10-20

**Authors:** Mi Jung Kwon, Ha Young Park, Joong Seob Lee, Eun Soo Kim, Nan Young Kim, Eun Sook Nam, Seong Jin Cho, Ho Suk Kang

**Affiliations:** 1 Department of Pathology, Hallym University Sacred Heart Hospital, Hallym University College of Medicine, Anyang 14068, Republic of Korea; mulank99@hallym.or.kr; 2 Department of Pathology, Busan Paik Hospital, Inje University College of Medicine, Busan 47392, Republic of Korea; mint@inje.ac.kr; 3 Department of Otorhinolaryngology-Head & Neck Surgery, Hallym University Sacred Heart Hospital, Hallym University College of Medicine, Anyang 14068, Republic of Korea; apniosio@hallym.or.kr; 4 Department of Radiology, Hallym University Sacred Heart Hospital, Hallym University College of Medicine, Anyang 14068, Republic of Korea; silwater007@hallym.or.kr; 5 Hallym Institute of Translational Genomics and Bioinformatics, Hallym University Medical Center, Anyang 14068, Republic of Korea; honeyny@hallym.or.kr; 6 Department of Pathology, Kangdong Sacred Heart Hospital, Hallym University College of Medicine, Seoul 05355, Republic of Korea; esnam@kdh.or.kr (E.S.N.); apilas@kdh.or.kr (S.J.C.); 7 Department of Internal Medicine, Hallym University Sacred Heart Hospital, Hallym University College of Medicine, Anyang 14068, Republic of Korea

**Keywords:** miRNAs, oropharynx, tonsil, squamous cell carcinoma, *TERT* promoter gene, hsa-miR-1285-5p, hsa-miR-663a

## Abstract

Tonsillar squamous cell carcinomas (TSCCs) exhibit high rates of human papillomavirus (HPV) positivity. The expression profiles of microRNA (miRNA), which are small RNA molecules that play pivotal roles in biological processes, in TSCC in relation to the HPV status and cancer-related genetic mutations are not well investigated. Herein, we expanded our previous research, which was focused on established clinicopathological and genetic mutational data, to profile miRNA expression in TSCC, aiming to identify clinically relevant targets for early diagnosis and therapeutic intervention. The miRNA profiles were analyzed using the nCounter Nanostring miRNA Expression assay in 22 surgically resected TSCC tissues and their contralateral normal tonsil tissues. The *TERT* promoter (*TERT*p) gene was the only relevant candidate gene associated with differentially expressed miRNAs in TSCC. Hierarchical clustering analysis revealed high expression levels of hsa-miR-1285-5p, hsa-miR-1203, hsa-miR-663a, hsa-miR-1303, hsa-miR-33a-5p, and hsa-miR-3615 coupled with low expression levels of hsa-miR-3182, hsa-miR-219a-2-3p, and hsa-miR-767-3p, which were associated with HPV-positive TSCC (*p* = 0.009). Functional enrichment analysis revealed that these dysregulated miRNAs tended to be involved in protein binding (molecular function) and cellular components (biological processes). Therefore, hsa-miR-1285-5p and hsa-miR-663a may be associated with HPV-positive *TERT*p-mutated tumors and may serve as potential treatment targets and biomarkers for early detection.

## 1. Introduction

Tonsillar squamous cell carcinomas (TSCCs) predominantly constitute the landscape of oropharyngeal cancers, accounting for 15–20% of all cases, and exhibit the highest rates of human papillomavirus (HPV) positivity among all oropharyngeal subsites [1,2]. Oropharyngeal cancers are linked to several risk factors, including HPV infection, alcohol consumption, tobacco use, exposure to environmental pollutants, and genetic factors [3]. The conventional treatment approach for locally advanced or regional tonsil cancers typically involves a combination of surgical intervention and radiotherapy or chemotherapy [4,5]. This therapeutic strategy stems from the relatively heightened sensitivity of TSCCs to chemotherapy and radiotherapy, accompanied by a generally more favorable prognosis and better response to radiochemotherapy among cases of HPV-positive TSCCs as opposed to non-HPV-related oropharyngeal cancers [2,5]. This distinction has even led to the modification of the staging system by the American Joint Committee on Cancer (AJCC), which now differentiates between HPV-positive and HPV-negative oropharyngeal cancers [6]. However, despite advancements, the unique anatomical features of tonsils often obscure tumors that grow beneath the surface mucosa, and the presence of intricate lymphatic vessel networks further complicates early detection [7]. Consequently, TSCCs often exhibit advanced stages and prompt metastasis upon diagnosis, surpassing the risks observed in other types of head and neck cancers [8,9], thus suggesting the need for urgent and effective diagnostic and therapeutic modalities.

Delineating between HPV-associated and HPV-negative head and neck squamous cell carcinomas (SCCs) reveals significant molecular disparities, ranging from genetic variations to epigenetic changes and distinct protein expression patterns [10,11]. These molecular distinctions highlight the unique attributes of HPV-positive SCCs, further reinforcing the stark contrast with HPV-negative SCCs [10,11,12]. We have recently conducted clinicopathological investigations focused on identifying clinically significant mutations and their association with prognosis and HPV infection in patients with TSCC [10,13]. We determined the prevalence of oncogenic/likely oncogenic mutations among 30 cancer-related genes, including *TP53*, *PIK3CA*, *PTEN*, *EGFR*, *SMAD4*, and *TERT* promoter (*TERT*p) genes, where mutations in *TP53*, receptor tyrosine kinase (RTK) pathway genes, and *TERT*p demonstrated prognostic relevance [10,13]. Moreover, emerging evidence suggests that the primary driving forces behind head and neck SCC may be epigenetic rather than genetic factors, highlighting the crucial involvement of miRNAs in modulating these epigenetic changes [11]. MircoRNAs (miRNAs), small RNA molecules, exert their effects by modulating gene expression through the inhibition of specific messenger RNAs (mRNAs) at the post-translational level [14]. Given their influence on pivotal biological processes, such as apoptosis, cellular proliferation, tumor development, and metastasis, miRNAs have emerged as central players in various physiological and pathological events [14], prompting us to expand our research to include further miRNA expression analysis.

Despite their significance, studies investigating miRNAs as potential biomarkers and therapeutic targets for oropharyngeal SCC, particularly TSCC, are limited. Only a limited number of published studies have highlighted specific miRNAs, such as miR-21 and miR-31, as candidates for diagnosis, prognosis, and treatment of oropharyngeal SCC, with an even more limited number of studies specifically focusing on tonsil cancer [15,16,17,18,19,20]. Furthermore, research exploring the association between miRNAs and cancer-related genes, particularly in the context of HPV-positive TSCC, remains scarce [21].

In this retrospective study, we sought to analyze miRNA expression profiling in TSCC, in relation to the HPV status and cancer-related genetic mutations, to identify clinically relevant targets for early diagnosis and therapeutic intervention.

## 2. Materials and Methods

### 2.1. Patients, Samples, and Clinicopathological and Genetic Mutational Data

A retrospective analysis of 80 consecutive patients with TSCC who underwent primary resection at Hallym University Sacred Heart Hospital between 1997 and 2018 was conducted using the electronic search of the Pathology Department database, considering patients without prior treatment and with comprehensive medical records available. These patients also participated in previous studies [10,13]. A total of 58 patients were excluded due to insufficient mutational results; unsuitability of formalin-fixed, paraffin-embedded (FFPE) tissue blocks; and the absence of contralateral normal tonsil tissues, which are required for molecular investigation of miRNA expression profiling. During the surgical removal of the primary tumor mass, contralateral normal tonsil tissues were also excised and pathologically confirmed to be devoid of tumor cells. Therefore, the final sample consisted of 22 TSCC samples and their corresponding contralateral normal tonsil tissue samples. As a result, 22 patients were included in this study; the related medical and radiological records and mutational results, and all hemotoxylin and eosin-stained slides, were reviewed, retrospectively. The use of contralateral normal tonsil tissues from the same patients served a dual purpose in our study, enabling both direct comparisons of miRNA expression profiles between tumor and normal tissues within the same genetic and environmental context and providing clinically relevant insights into molecular alterations specific to TSCC [22].

The patient staging was updated according to the 8th edition of the Union for International Cancer Control (UICC) and the AJCC (AJCC/UICC) TNM classification [6]. Heavy smoking was categorized as a smoking history exceeding 20 pack-years, while excessive alcohol consumption was classified as consuming more than 14 drinks per week [6]. The present study received ethical approval from the Institutional Review Board of Hallym University Sacred Heart Hospital (HALLYM 201fff-012) and was conducted in accordance with the principles outlined in the Declaration of Helsinki.

The previously established data of next-generation sequencing (NGS) were retrieved, and NGS was previously performed using the Ion Personal Genome Machine Sequencer tool (Thermo Fisher Scientific Libraries for each sample were prepared using the Ion AmpliSeq Library Kit 2.0 and Ion AmpliSeq Cancer HotSpot Panel v2, both from Thermo Fisher Scientific, Waltham, MA, USA) following the manufacturer’s guidelines and previously described methods [10]. Variant calling was performed using Ion Torrent platform-specific software, and annotation was performed using the Variant Effect Predictor. We cross-referenced our data with findings from other cancer genome studies via cBioPortal and confirmed hotspot mutations. Raw annotated variants were screened for probable somatic variants based on specific criteria, manually reviewed, and visually validated using the Integrated Genomics Viewer. Real-time peptide nucleic acid-mediated PCR or sequencing methods were used for validation. HPV status was assessed using the PANA RealTyper HPV Genotyping Kit (PANAGENE, Daejeon, Republic of Korea) as previously described [10].

### 2.2. MicroRNA Extraction and Nanostring nCounter miRNA Expression Assay

miRNAs were extracted from FFPE tissues using the miRNeasy Mini Kit (Qiagen, Hilden, Germany) following the manufacturer’s protocol. The sample concentrations were determined using a NanoDrop spectrophotometer (Thermo Fisher Scientific). This study utilized nCounter human v3 miRNA expression assays (Nanostring Technologies, Seattle, WA, USA). The miRNA panel contained oligonucleotide tags linked to 798 human miRNAs (sourced from miRBase v21) and 5 reference housekeeping mRNAs (ACTB, B2M, GAPDH, RPL19, and RPLP0). The performance and specificity of each reaction stage were monitored using 25 control probes to detect synthetic mRNA or miRNA targets. For the hybridization step (16 h at 65 °C), 150 ng of total RNA was combined with probe pairs, which included reporter probes with a signal at their 5′ end and capture probes with biotin at their 3′ end. After hybridization, we performed sample clean-up and produced digital count reports following the manufacturer’s guidelines.

The initial data were analyzed using the Nanostring nSolver (version 4.0). To ensure accurate and reliable analysis, we performed quality control (QC) on the raw data using Nanostring QC Pro version 1.14.0. The QC was conducted based on four parameters: (1) imaging QC, which required the field of view to be over 75%; (2) binding density QC, where the binding density value should be between 0.1 and 2.25; (3) positive control linearity QC, necessitating an R2 value above 0.95; and (4) a limit of detection QC, stipulating that the 0.5 fM positive control probe must exceed two standard deviations plus or minus the average of the negative controls. To normalize the raw data, we employed the spike-in method, which uses synthetic spike-in control RNA targets to compensate for any variance introduced during sample purification.

### 2.3. Function Enrichment Analysis

Gene Ontology (GO) provides a consolidated database of digital information related to gene functions [23]. The GO annotations were divided into three categories: (1) biological processes, (2) cellular components, and (3) molecular functions. The Kyoto Encyclopedia of Genes and Genomes (KEGG) provides high-level functional interpretations and practical applications of genomic data [24]. An overlap test was conducted to identify the functional biological implications of genes expressed differently between the compared biological conditions. This test was performed between differentially expressed genes and those classified by function, including both KEGG pathways and GO. G:Profiler (https://biit.cs.ut.ee/gprofiler/gost, accessed on 3 June 2023) was used for the statistical enrichment of functional annotations. Furthermore, differentially expressed genes with a prediction score of 80 or above were targeted using miRDB (https://mirdb.org, accessed on 25 February 2023) to predict miRNA gene targets.

### 2.4. Statistical Analyses

To identify differential gene expression between mutational tumor tissues and normal control tissues, we utilized nSolver (version 4.0) and applied the two-stage Benjamini, Krieger, and Yekutieli procedure with a false discovery rate (FDR) threshold set at less than 0.05 (https://www.nanostring.com/products/analysis-software/nsolver, accessed on 5 June 2023). Unsupervised clustering of the normalized expression values of the selected differentially expressed miRNAs across samples was conducted using custom R scripts to analyze expression profiles. Scatter plots illustrating gene expression values against expression *p*-values between the two chosen samples were generated using these R scripts. The top five considerably enriched GO terms for the selected target genes were determined by GO term enrichment analysis based on adjusted *p*-values using the Benjamini–Hochberg procedure [25]. Statistical significance was set at *p* < 0.05.

## 3. Results

### 3.1. Baseline Characteristics

The clinical and pathological data and molecular profiling data are summarized in Table 1. The most common mutations were *TP53* (63.6%, 14/22), followed by *PIK3CA* (45.5%, 10/22), *PTEN* (27.3%, 6/22), *SMAD3* (22.7%, 5/22), *EGFR*, and *RB1* (each 18.2%, 4/22), with other mutations distributed across several other genes (*RAS* (13.6%), *FBXW7* (13.6%), *TERT*p (9.1%), *SMARCB1* (9.1%), *PDGFRA* (9.1%), *CDKN2A* (9.1%), *KIT* (9.1%), *HRAS* (4.5%), *NOTCH1* (4.5%), *IDH1* (4.5%), *STK11* (4.5%), *HER2* (4.5%), *FGFR2* (4.5%), *FLT3* (4.5%), *AKT1* (4.5%), *RET* (4.5%), *IDH2* (4.5%), *KRAS* (4.5%), *CDH1* (4.5%), *ATM* (4.5%), and *ALK* (4.5%)). The study included patients with HPV-positive (77.3%, 17/22) and HPV-negative (22.7%, 5/22) tumors. Among the patients with HPV-positive tumors, the majority (n = 14) harbored HPV genotype 16, whereas three harbored HPV genotype 18. The HPV-positive patients significantly outperformed their HPV-negative counterparts in terms of the 5-year overall survival rate (*p* = 0.010) and 5-year disease-free survival rate (*p* = 0.035).

### 3.2. Screening of Candidate miRNAs Associated with Specific Genetic Mutations

**Figure 1:**
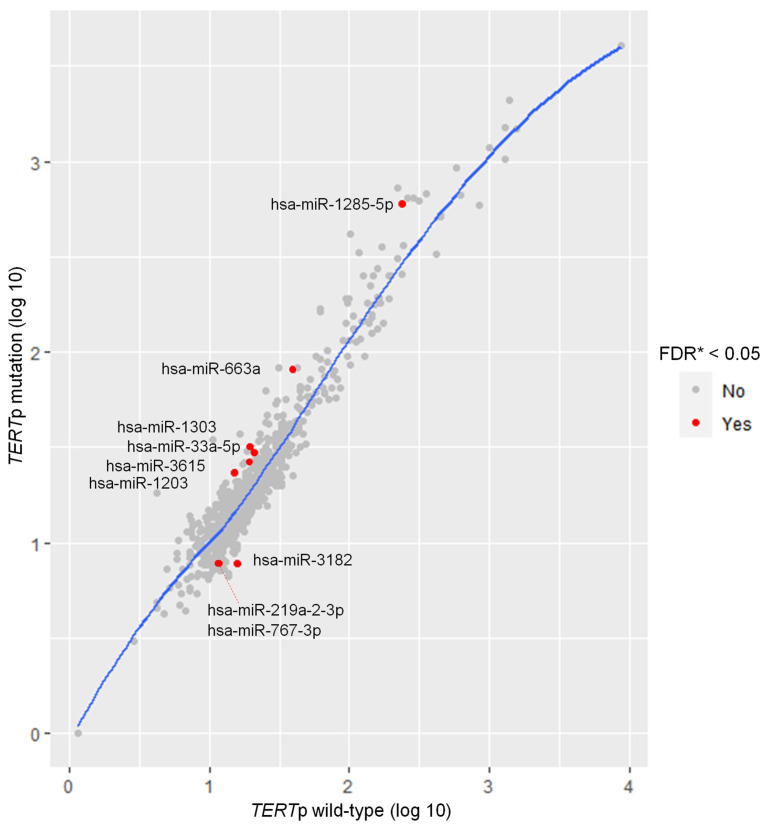
Scatter plot analysis of miRNA expression comparisons in tonsillar squamous cell carcinomas: *TERT*p-wild-type versus *TERT*p mutation. Significance was determined using the two-stage method of Benjamini, Krieger, and Yekutieli. * Significant, FDR < 0.05.

Our analysis identified 798 miRNAs associated with 13 established genetic mutations in the 22 TSCC samples. The results revealed *TERT*p mutation to be the only mutation of interest specifically associated with differentially expressed miRNAs among the analyzed genetic mutations. Upon comparing the miRNA expression profiles of 20 *TERT*p wild-type samples and two *TERT*p-mutated samples, we identified six differentially expressed miRNAs (hsa-miR-1285-5p, hsa-miR-1203, hsa-miR-663a, hsa-miR-1303, hsa-miR-33a-5p, and hsa-miR-3615) with upregulated expression and three miRNAs (hsa-miR-3182, hsa-miR-219a-2-3p, and hsa-miR-767-3p) with downregulated expression in *TERTp*-mutated tumors, with all results exhibiting an FDR of less than 0.05 (Table 2 and Figure 1).

Our comparison of miRNA expression patterns between *TERT*p-mutated samples and normal tonsil tissue samples also indicated that two miRNAs (hsa-miR-1285-5p and hsa-miR-663a) were significantly upregulated in *TERT*p-mutated tumors.

**Figure 2:**
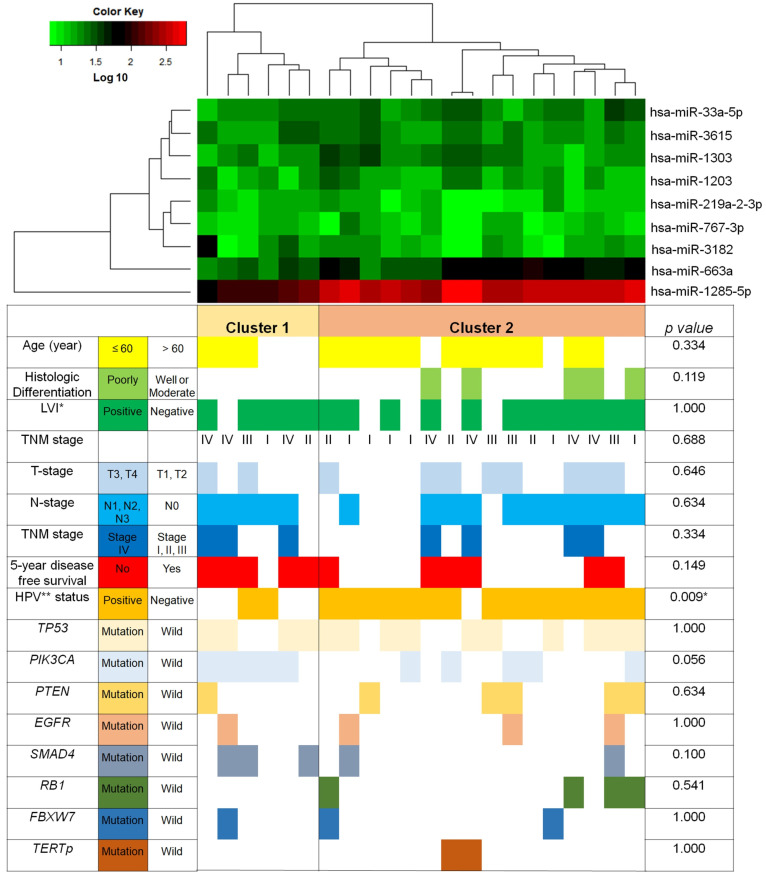
Hierarchical clustering analysis of differentially regulated miRNAs in tonsillar squamous cell carcinomas: TERTp wild-type versus TERTp mutation. Each row corresponds to a specific miRNA, while each column represents an individual sample in the analysis. * LVI: lymphovascular invasion, ** HPV: human papillomavirus. (* Significant, *p* < 0.05).

We performed an unsupervised hierarchical clustering analysis to examine the expression patterns of the nine dysregulated miRNAs (Figure 2). This analysis divided the tumor samples into two primary clusters. Cluster 2 exhibited greater expression levels of the six upregulated miRNAs and lower expression levels of the three downregulated miRNAs than Cluster 1. Notably, Cluster 2 had a significantly higher association with HPV positivity than Cluster 1 (HPV-positivity Cluster 1 (2/6, 33.3%) vs. Cluster 2 (15/16, 93.8%); *p* = 0.009). In contrast, Cluster 1 was predominantly HPV-negative and exhibited a tendency toward association with *PIK3CA* mutation, although this association was not statistically significant (*p =* 0.056).

### 3.3. Identification of Potential Functional Pathways Related to miRNAs

**Figure 3:**
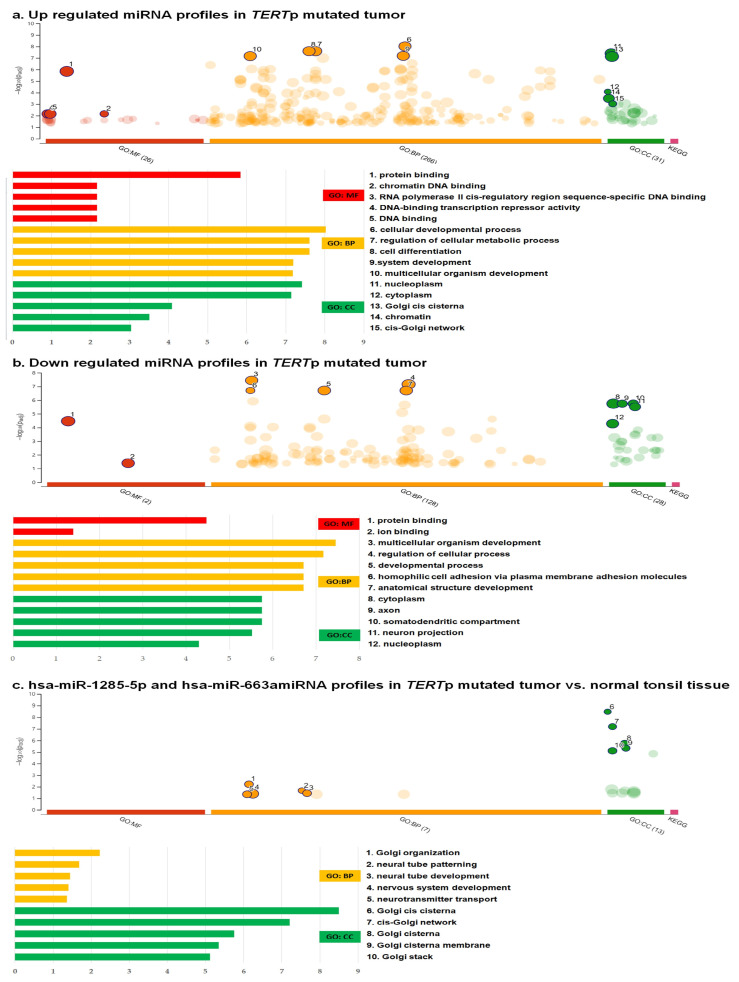
Top five Gene Ontology (GO) processes, ranked based on their enrichment scores (−log10 adjusted *p*-values using the Benjamini–Hochberg procedure), were identified for two sets of miRNAs. The first set (**a**) included six upregulated miRNAs (hsa-miR-1285-5p, hsa-miR-1203, hsa-miR-663a, hsa-miR-1303, hsa-miR-33a-5p, and hsa-miR-3615), whereas the second set (**b**) consisted of three downregulated miRNAs (has-miR-3182, hsa-miR-219a-2-3p, ahashsa-miR-767-3p) in *TERT*p-mutated tumors. Additionally, two upregulated miRNAs (hsa-miR-1285-5p and hsa-miR-663a) were compared with normal tonsil tissues (**c**). The functional terms were derived from various sources, including the Kyoto Encyclopedia of Genes and Genomes (KEGG) pathway, the biological process (BP) category of GO, the cellular component (CC) category of GO, and the molecular function (MF) category of GO.

To ascertain the biological implications of the six upregulated and three downregulated miRNAs that displayed significant differences in expression profiles associated with *TERT*p mutation, we performed GO analysis. The top five GO processes, sorted by enrichment score (−log10(*p*-value)), were itemized for the upregulated and downregulated miRNAs (Figure 3a,b). In the GO analysis, for the upregulated miRNAs, the most enriched GO functional annotation revealed cellular biological processes that involve protein binding, chromatin DNA binding, RNA polymerase II cis-regulatory region sequence-specific DNA binding, DNA-binding transcription repressor activity, and DNA binding in molecular functions; cellular developmental process, regulation of cellular metabolic process, cell differentiation, system development, and multicellular organism development in biological processes; and nucleoplasm, cytoplasm, Golgi cis-cisterna, chromatin, and cis-Golgi network in cellular components. Moreover, the highest-ranking GO processes associated with the downregulated miRNAs included protein binding and ion binding in molecular functions and multicellular organism development, regulation of cellular processes, developmental processes, homophilic cell adhesion via plasma membrane adhesion molecules, anatomical structure development, and cytoplasm in biological processes. The implicated cellular components were the axon, somatodendritic compartment, neuron projections, and nucleoplasm.

KEGG pathway analysis was conducted to understand the molecular interactions and pathways associated with these genes; however, no significant results were obtained. For the six upregulated miRNAs, the top 10 enriched KEGG pathways were related to fatty acid metabolism, calcium signaling pathway, AGE-RAGE signaling pathway in diabetic complications, fatty acid degradation, neurotrophin signaling pathway, renin secretion, transcriptional misregulation in cancer, hepatitis B, cell cycle, and MAPK signaling pathway. In contrast, for the three downregulated miRNAs, the top 10 enriched KEGG pathways were the hedgehog signaling pathway, Wnt signaling pathway, Hippo signaling pathway, circadian rhythm, cancer pathways, selenocompound metabolism, insulin signaling pathway, Th17 cell differentiation, cellular senescence, and spinocerebellar ataxia.

The GO and KEGG pathway analysis results on the biological implications of the upregulated hsa-miR-1285-5p and hsa-miR-663a were similar between *TERT*p-mutated tumors vs. normal tonsil tissues (Figure 3c).

## 4. Discussion

In this study, we analyzed the expression levels of 798 miRNAs in tonsil cancers and identified *TERT*p as a candidate gene that showed a specific association with miRNAs included. *TERT*p, a critical regulatory element involved in controlling telomerase expression with binding sites for numerous transcriptional activators and repressors, contributes to enhanced telomerase activity, leading to cellular immortalization, a distinct feature of cancer [26,27]. Mutations at nucleotides 124 and 146 in the core promoter region of *TERT* are pathogenic mutations in human malignancies [28] and are driving mutations in head and neck SCCs [12,29,30,31]. A thorough meta-analysis suggested that *TERT*p mutations represent a poor prognostic marker across all types of cancers, regardless of their origin, including head and neck cancers [12,30,31,32]. In line with this study, our previous study revealed that *TERT*p mutations independently serve as unfavorable prognostic indicators for disease-free survival among patients with TSCC when evaluated using the eighth updated staging system, reinforcing the clinical importance of *TERT*p mutations in tonsil cancer [13]. In the present study, using miRNA expression profiling, we expanded our results to include epigenetic regulation of *TERT*p in tonsil cancers.

To date, only a few studies have analyzed miRNA expression in HPV-related and HPV-unrelated oropharyngeal cancers [15,33,34], where no commonly shared miRNA dysregulations have been identified across studies [15,33,34]. While previous investigations have examined the presence of *TERT*p mutations in various subsites of oral and oropharyngeal cancers [35,36], specific miRNAs linked to *TERT*p mutations in tonsil cancers and their correlation with HPV have been sparingly explored. In the present study, we identified a correlation between *TERT*p mutations and a distinct differential expression pattern: upregulation of six miRNAs (hsa-miR-1285-5p, hsa-miR-1203, hsa-miR-663a, hsa-miR-1303, hsa-miR-33a-5p, and hsa-miR-3615) and downregulation of three miRNAs (hsa-miR-3182, hsa-miR-219a-2-3p, and hsa-miR-767-3p) in tonsil cancers. Although the miRNAs hsa-miR-663 and hsa-miR-219 reported in our study have been also previously reported in tonsil cancers [18], the previous study did not explore their clinical and pathological relevance in tonsil cancers [18]. Given that *TERT*p mutations, both individually and in conjunction with HPV oncogenes, significantly affect oral and uterine cervical SCCs [29,37] and that a major part (83.3%) of observed *TERT*p mutations has been detected in HPV-related TSCCs [13], this differential expression pattern may be used to distinguish between two clinically relevant groups: HPV-positive and HPV-negative tonsil cancers. Moreover, two viral oncogenes, E6 and E7, are associated with high-risk HPV-related cancers and influence the oncogenic pathway, leading to cellular immortalization, particularly by promoting telomerase expression, which may contribute to unfavorable prognosis [13,38,39]. Therefore, highly expressed miRNAs specific to HPV-positive TSCC with *TERT*p mutations, considered unfavorable prognostic indicators, may hold clinical significance in the context of the current updated AJCC staging system distinguishing between HPV-positive and HPV-negative oropharyngeal cancers [6].

In addition, a notable finding of our present study is that both hsa-miR-1285-5p and hsa-miR-663a exhibited substantial upregulation within *TERT*p-mutated tumors in contrast to normal tonsil tissues and *TERT*p wild-type tumors. This observation may indicate their potential involvement in the initiation and progression of tumorigenesis within tonsil cancer. Moreover, the elevated expression of these miRNAs in *TERT*p-mutated tumors may hint at their potential clinical utility as markers for early detection of tonsil cancer. The intricate involvement of miR-1285-5p and miR-663a in diverse cancer contexts highlights their multifaceted contributions to the intricacies of the development and progression of various cancers. Moreover, the increased expression of miR-1285-5p in non-small cell lung cancer tissues suggests its role as a potential promoter of tumor development, regulating critical behaviors, such as proliferation, invasion, and migration through interactions with genes, such as *Smad4* and *CDH1* [40]. In thyroid cancer, increased expression of miR-1285-5p correlates with the invasive growth of the follicular variant of papillary thyroid carcinomas [41]. Similarly, miR-663a has demonstrated upregulated expression across diverse cancer types, such as nasopharyngeal carcinoma, lung cancer, and colon cancer, reflecting its versatile functions in distinct biological contexts and its modulation of pivotal genes, such as *p21*, *TGF-β1*, and *CXCR4-p21* [42,43,44]. Conversely, in certain cancers, miR-1285-5p and miR-663 have been implicated in contrasting mechanisms. While miR-1285-5p acts as a tumor suppressor in renal cell carcinoma, its diminished expression promotes tumor progression [45]. In gastric cancer, reduced miR-663 levels may contribute to abnormal cell hyperplasia and cancer progression [46]. These phenomena may be explained by the fact that miRNAs can uniquely control a multitude of protein-coding genes, and their expression patterns can vary among different organs and tissues [40,41,42,43,44,45]. Nevertheless, few studies have focused on the role of hsa-miR-1285-5p or hsa-miR-663a in tonsil cancers, and the present study is the first to report the upregulation of hsa-miR-1285-5p and hsa-miR-663a in clinical TSCC samples, in terms of *TERT*p mutation. Thus, identifying novel molecular targets regulated by miR-1285-5p and miR-663 may be crucial to enhancing our understanding of tonsil cancer oncogenesis and treatment.

In our study, we conducted a comprehensive GO analysis to uncover the functional implications of the *TERT*p mutation in conjunction with the identified miRNAs. Notably, our GO analysis highlighted a common theme across all nine miRNAs, namely, their involvement in protein binding and cellular component processes. Particularly noteworthy were the associations with cytoplasmic organelles, with a specific focus on the Golgi apparatus [47]. Among the miRNAs under investigation in the present study, hsa-miR-1285-5p and hsa-miR-663 exhibited significant enrichment in the cellular components of the Golgi apparatus when compared to levels in normal tonsil tissues. This enrichment was confirmed by a high level of significance in the GO analysis. The Golgi apparatus, a crucial organelle situated in the cytoplasm near the endoplasmic reticulum and adjacent to the cell nucleus, plays a pivotal role in facilitating the transport of proteins and lipids within eukaryotic cells [47]. Importantly, previous research has highlighted the intricate web of protein-coding genes targeted by miR-663a [48]. In the context of renal cell carcinoma, increased expression of miR-663a has been reported to influence cellular functions [48]. Building upon these insights, our study may further implicate miR-663a, along with miR-1285-5p, in the regulatory processes of the Golgi apparatus, potentially affecting the intricate machinery governing cellular transport and communication. Although our KEGG pathway analysis revealed several signaling and cancer-related pathways, none reached statistical significance. This observation might indicate that these miRNAs, in collaboration with the *TERT*p mutation, may primarily influence the upstream regulation of cellular biological components, rather than being directly linked to specific signaling pathways, suggesting the complexity of miRNA-mediated mechanisms and their potential impact on fundamental cellular processes.

Our study has several limitations that need to be acknowledged. First, the number of samples was relatively low, and exclusively male participants were included in this pilot study, and a broader population is required in further investigations. Second, we did not validate our results using quantitative PCR. However, we utilized Nanostring technology to detect cancer-related miRNAs among a pool of 798 miRNAs, eliminating the need for transcript amplification. This approach allowed us to identify potentially low-abundance transcripts that could have posed challenges for validation via PCR [49]. Nonetheless, our study may contribute to the understanding of the correlation between *TERT*p mutations and miRNA profiles in tonsil cancers, a field that is not yet well elucidated. The findings of our study may highlight the association of hsa-miR-1285-5p and hsa-miR-663a with HPV-positive and *TERT*p-mutated tumors compared to normal tonsil tissues, which may thus be used as potential screening markers for early detection.

In summary, we identified differentially expressed miRNAs associated with *TERT*p mutation in tonsil cancers, which may have the clinical potential to serve as candidate biomarkers for early detection through screening efforts and as targets for precision medicine-based therapeutic interventions. 

## Figures and Tables

**Table 1:** Baseline characteristics in tonsil cancers.

Characteristic	n = 22 (%)	HPV-Negative n = 5 (22.7%)	HPV-Positive n = 17 (77.3%)	*p*-Value
Sex				
Male	22 (100)	5 (100)	17 (100)	
Female	0 (0.0%)			
Age, mean ± SD (years)	55.22 ± 9.35 (range, 41–73)			
≤60	15 (68.2)	3 (60.0)	12 (70.6)	0.655
>60	7 (31.8)	2 (40.0)	5 (29.4)	
Smoking				
Light	7 (31.8)	1 (20.0)	6 (35.3)	0.519
Heavy	15 (68.2)	4 (80.0)	11 (64.7)	
Alcohol				
Light	11 (50)	1 (20.0)	10 (58.8)	0.127
Heavy	11 (50)	4 (80.0)	7 (41.2)	
Lymphovascular invasion				
negative	5 (22.7)	1 (20.0)	4 (23.5)	
positive	17 (77.3)	4 (80.0)	13 (76.5)	1000
pT category				
T1–T2	12 (54.5)	4 (80.0)	8 (47.1)	0.193
T3–T4	10 (45.5)	1 (20.0)	9 (52.9)	
pNodal status				
N0–1	13 (59.1)	1 (20.0)	12 (70.6)	0.116
N2–3	9 (40.9)	4 (80.0)	5 (29.4)	
AJCC stage				
I–II	15 (68.2)	1 (20.0)	10 (58.8)	0.311
III–IV	7 (31.8)	4 (80.0)	7 (41.2)	
5-year overall survival				
Survival	12 (54.5)	0 (0.0)	12 (70.6)	0.010 *
Death	10 (45.5)	5 (100.0)	5 (29.4)	
5-year disease-free survival				
Yes	11 (50.0)	0 (0.0)	11 (64.7)	0.035 *
No	11 (50.0)	5 (100.0)	6 (35.3)	
Mutated detected tumors				
No detected	0 (0.0)	0 (0.0)	0 (0.0)	
Detected	22 (100)	5 (100.0)	17(100.0)	
*TP53*				
Wild type	8 (36.4)	0 (0.0)	8 (47.1)	0.054
Mutated	14 (63.6)	5 (100.0)	9 (52.9)	
*PICK3A*				
Wild type	12 (54.5)	2 (40.0)	10 (58.8)	0.457
Mutated	10 (45.5)	3 (60.0)	7 (41.2)	
*PTEN*				
Wild type	16 (72.7)	4 (80.0)	12 (70.6)	0.678
Mutated	6 (27.3)	1 (20.0)	5 (29.4)	
*SMAD4*				
Wild type	17 (77.3)	4 (80.0)	14 (82.4)	0.905
Mutated	5 (22.7)	1 (20.0)	3 (17.6)	
*EGRF*				
Wild type	18 (81.8)	3 (60.0)	14 (82.4)	0.294
Mutated	4 (18.2)	2 (40.0)	3 (17.6)	
*TERT*p				
Wild type	20 (90.9)	4 (80.0)	16 (94.1)	0.334
Mutated	2 (9.1)	1 (20.0)	1 (5.9)	

HPV, human papillomavirus; SD, standard deviation; AJCC, American Joint Committee on Cancer. * Significant, *p* < 0.05.

**Table 2:** Comparative miRNA expression profiles between *TERT*p-mutated tonsillar squamous cell carcinoma (TSCC) and wild-type TSCC and between *TERT*p-mutated TSCC and normal control tonsil tissues.

	*TERT*p*-*Mutated vs. Wild Type		Expression in Mutated Tumors	*TERT*p-Mutated vs. Normal Control		Expression in Mutated Tumors
	Fold Change	FDR *		Fold Change	DE Call *	
hsa-miR-1285-5p	2.53	0.00	up	3.13	Yes	up
hsa-miR-1203	1.56	0.01	up	2.83	No	up
hsa-miR-3182	−2.06	0.01	down	−1.35	No	down
hsa-miR-663a	2.02	0.01	up	7.31	Yes	up
hsa-miR-219a-2-3p	−1.57	0.01	down	−1.29	No	down
hsa-miR-767-3p	−1.52	0.01	down	−1.72	No	down
hsa-miR-1303	1.63	0.02	up	1.72	No	up
hsa-miR-33a-5p	1.42	0.04	up	−2.4	No	down
hsa-miR-3615	1.31	0.04	up	2.7	No	up

Significance was determined by nSolver’s differential expression (DE) call function and the two-stage method of Benjamini, Krieger, and Yekutieli. * Significant, FDR < 0.05. FDR, false discovery rate.

## Data Availability

The data used to support the findings of this study are available from the corresponding author upon request.
